# Supplementary material for: Prolonged standing reduces fasting plasma triglyceride but does not influence postprandial metabolism compared to prolonged sitting
Source: PLoS One. 2020 Feb 5;15(2):e0228297. doi: 10.1371/journal.pone.0228297 (PMC7001955; doi:10.1371/journal.pone.0228297)
Supplement: S1 Table — This shows the protocol as approved by the University of Texas at Austin’s Institutional Review Board. (PDF) [file pone.0228297.s001.pdf]

IRB USE ONLY
Study Number: 2016-12-0031
Approval Date: 03/03/2017
Expires: 03/02/2018

Consent for Participation in Research

Title: *The Effects of Prolonged Standing Compared to Prolonged Sitting on Postprandial Lipemia*

Principal Investigator(s), UT affiliation, and Telephone Number(s):

Charles Kikutaro Crawford, B.S. – Department of Kinesiology and Health Education - Phone: (817) 781-5117, email: kikicrawford92@utexas.edu

Edward F. Coyle, Ph.D. Professor – Department of Kinesiology and Health Education- Phone: (512) 471-8596, email: coyle@austin.utexas.edu.

Funding source: Partial funding from a faculty grant from UT-Austin

Introduction

You are being asked to participate in a research study. This form provides you with information about the study. The Principal Investigator (the person in charge of this research) or his/her representative will provide you with a copy of this form to keep for your reference, and will also describe this study to you and answer all your questions. Please read the information below and ask questions about anything you don't understand before deciding whether to take part. Your participation is entirely voluntary and you can refuse to participate without penalty or loss of benefits to which you are otherwise entitled.

What is the purpose of this study?

The purpose of this study is to investigate the effect that 12 hours of prolonged standing will have on lipid metabolism compared to prolonged sitting.

What will you be asked to do?

If you agree to take part in this study, you will first be asked to fill out a health questionnaire and have your weight and height measured. If you are apparently healthy and at low risk for cardiovascular disease you will be invited to participate.

You will be asked to partake in preliminary testing to determine your metabolic rate while sitting and standing. You will then go through two trials; each trial will consist of three phases: a controlled activity phase, an intervention phase, and a high fat tolerance test phase. The only difference between these trials will be the intervention phase of either 12 hours of sitting or 12 hours of standing. Each trial will be separated by at least 7 days.

Each trial will consist of four days with three visits to the Human Performance Laboratory
Trial 1: Plasma triglyceride responses with an intervention of 12 hours of sitting.
Trial 2: Plasma triglyceride responses with an intervention of 12 hours of standing.

The order of the trials will be randomized.

Step-by-Step Protocol:

- **Preliminary Testing**
  1. Arrival (10:00)
  2. Read and sign informed consent (10:05)
  3. Fill out health history questionnaire (10:15)
  4. Measurement of height and weight (10:25)
  5. Seated metabolic measurement (10:30)
     - You will sit down for 30 minutes while breathing into a mask that will measure your expired gases to determine your metabolic rate.
  6. Standing metabolic measurement (11:00)
     - You will stand for 30 minutes while breathing into a mask that will measure your expired gases to determine your metabolic rate.

Total time in Preliminary Testing: Approximately 1.5 hours

- **Controlled Activity Phase**
  1. Arrival (8:00)
  2. Installation of pedometer (8:05)
     - You will wear a pedometer on your waist and use the visual feedback to ensure that you are taking the correct number of steps.
  3. Installation of activPAL activity monitor (8:10)

    - A small flat device will be placed on your right thigh and secured with Tegaderm tape. You will wear this continually.
4. Verbal explanation of restricted activities, food log, and sleep log (8:15)
    - You will be asked to take between 5,5000 and 6,5000 steps per day while away from the laboratory.
    - You will be asked to document all consumed foods into a provided log, as well as take pictures of what you eat.
    - You will be asked to record the time of day that you go to sleep.
    - You will be asked to wear a waterproof cast cover on your right leg when you shower. This will be provided by the laboratory.

Total time in Controlled Activity Phase: Approximately 40 minutes (20 minutes x 2 visits)

- **<u>Sit/Stand Intervention Phase</u>**

1. Arrival (8:00)
2. Removal of activPAL activity monitor and pedometer (8:05)
3. Installation of new activPAL activity monitor (8:10)
4. Breakfast (8:30)
5. Prolonged sitting/standing (9:00-21:00)
    - You will be asked to either sit or stand still for 12 hours. For standing, you will be given a 15-minute break to sit down while you eat your snack at 17:00.
6. Lunch (12:00)
7. Snack (17:00)
8. Dinner (21:00)
9. Installation of pedometer (during second intervention, to allow visual feedback for subject to replicate step count from previous visit) (21:05)
    - On your second trial, you will be given a pedometer to wear on your waist. You will be instructed to take a certain number of steps during your time away from the laboratory. This will equal the number of steps that you took after your first trial.
    - During your time away from the laboratory you will be asked to minimize sitting and standing, and to lay down as much as possible.

Total time in Intervention Phase: Approximately 26 hours (13 hours x 2 visits)

- **<u>High Fat Tolerance Test Phase</u>**

1. Arrival (8:00)

2. activPAL and pedometer removal (8:05)
3. Bodyweight measurement (8:10)
4. Catheter insertion (8:15)
   - A catheter will be inserted into a vein near your elbow by a certified phlebotomist.
   - The catheter is a thin and flexible tube that will be inserted into a large arm vein. It will be secured to the arm and remain in the vein for the entirety of the test and will be removed upon collection of the final sample. It should cause no discomfort after insertion.
5. Fasted blood sample collection (8:20)
   - All collected blood samples will contain 6 mL pulled from the catheter.
6. High fat shake ingestion (8:25)
   - You will be given a shake made of melted ice cream and dairy creamer. You will be asked to consume the shake within five minutes.
7. Hourly postprandial blood samples (9:30, 10:30, 11:30, 12:30, 13:30, 14:30)
8. Expired gas collection at 1, 3, 5 hours (9:30, 11:30, 13:30)
   - While seated, you will be asked to breath into a mask to measure your expired gases for 20 minute periods.

Total time in High Fat Tolerance Test Phase: Approximately 14 hours (7 hours x 2 visits)
<u>Total Time of Involvement: Approximately 42 hours, 10 minutes</u>

What are the risks involved in this study?
None of these procedures are expected to be unduly painful in a healthy individual. Blood samples will be collected during both high fat tolerance tests with a venous catheter in a vein located near your elbow. There is a risk of infection when using catheters, but the area will be cleaned with an alcohol wipe prior to insertion to minimize this risk. Minor pain or discomfort may occur during the insertion of the catheter; the feeling associated with catheter insertion is similar the that obtained during a blood draw at a doctor's office and may result in slight bleeding, pain, and swelling of the vein. After the catheter has been placed, a saline flush will be attached to prevent clogging of the line in between blood draws. The total amount of blood drawn per test will be 84 mL, which is equal to about 6 tablespoons. This amounts to less than 1.5% of your total blood volume.

The activity monitor that you will wear on your right thigh is small, and should not prevent you from doing non-aquatic activities.

Potential risks of prolonged standing include fainting and joint discomfort. If at any time you feel like you may faint, you should immediately lay down as well as tell one of the investigators. You are encouraged to occasionally shift your weight back and forth to activate skeletal muscle pumps, which will help move blood out of your legs. You will also be standing on a cushioned mat, to ease joint discomfort.

You are free to discontinue your participation in this study at any time and for any reason. There may also be additional risks that are not currently known. If you wish to discuss any of the information above or any potential risks, please feel free to ask questions now or call the Principal Investigators listen on the first page of this form.

What are the possible benefits of this study?

You will be provided with a graphic and verbal description of your metabolic rate. You will also be given a graphic and verbal description of your plasma triglyceride concentration, which is indicative of your health. If any values are abnormal, you will be advised to see your physician.

Do you have to participate?

No, your participation is voluntary. You may decide not to participate at all or, if you start the study, you may withdraw at any time and for any reason. Withdrawal or refusing to participate will not affect your relationship with The University of Texas at Austin or the Principal Investigators in anyway.

To withdraw from this study please contact one of the principal investigators: Edward F. Coyle at (512) 471-8596 or Charles K. Crawford at (817) 781-5117. You should also feel free to call for any questions, concerns, or complaints about the research.

If you would like to participate, please give a signed copy of this form to one of the principal investigators. You will receive a copy of this form for your own personal records.

What are the alternatives to participating in this research?

Your participation is voluntary, and there is no consequence for withdrawing or refusing to participate in this research.

Will there be any compensation?

You will not receive any type of payment for participating in this study.

What if you are injured because of the study?

1. The University has no program or plan to provide treatment for research related injury or payment in the event of a medical problem. In the event of a research related injury, please contact the principal investigator.

2. The University has no program or plan for continuing medical care and/or hospitalization for research-related injuries or for financial compensation.

3. If injuries occur as a result of study activity, eligible University students may be treated at the usual level of care with the usual cost for services at the Student Health Center, but the University has no program or plans to provide payment in the event of a medical problem.

How will your privacy and confidentiality be protected if you participate in this research study?

You will be assigned a unique Subject ID code. This informed consent form and the Health History Questionnaire are the only places where any personal identifying information will be recorded. These forms will be stored in a locked file cabinet. In all other cases, your data will only be identifiable by your unique code. Only the directors of the project will have access to a master list that will link your identity to your code.

If in the unlikely event it becomes necessary for the Institutional Review Board to review research records, then the University of Texas at Austin will protect the confidentiality of your records to the extent permitted by law. Your research records will not be released without your consent unless required by law or a court order. The data resulting from your participation may be made available to other researchers in the future for research purposes not detailed within this consent form. In these cases, the data will contain no identifying information that could associate you with it, or with your participation in any study.

If the results of this research are published or presented at scientific meetings, your identity will not be disclosed.

Whom to contact with questions about the study?

Prior, during or after your participation you can contact the researcher, Edward F. Coyle at (512) 471-8596 or coyle@austin.utexas.edu.You may also contact Charles K. Crawford at (817) 781-5117 or

kikicrawford92@utexas.edu. Please contact them for any questions or if you feel that you have been harmed.

Whom to contact with questions concerning your rights as a research participant?

For questions about your rights or any dissatisfaction with any part of this study, you can contact, anonymously if you wish, the Institutional Review Board by phone at (512) 471-8871 or email at orsc@uts.cc.utexas.edu This study has been reviewed and approved by The University Institutional Review Board and the study number is 2016-12-00031.

Participation

If you agree to participate, please turn in a signed copy of this form to Charles K. Crawford or Edward F. Coyle.

Signature

You have been informed about this study's purpose, procedures, possible benefits and risks, and you have received a copy of this form. You have been given the opportunity to ask questions before you sign, and you have been told that you can ask other questions at any time. You voluntarily agree to participate in this study. By signing this form, you are not waiving any of your legal rights.

____

Printed Name

___________________________________ __________________

Signature Date

As a representative of this study, I have explained the purpose, procedures, benefits, and the risks involved in this research study.

____

Print Name of Person obtaining consent

___________________________________ __________________

Signature of Person obtaining consent Date
