# Supplementary material for: Prolonged standing reduces fasting plasma triglyceride but does not influence postprandial metabolism compared to prolonged sitting
Source: PLoS One. 2020 Feb 5;15(2):e0228297. doi: 10.1371/journal.pone.0228297 (PMC7001955; doi:10.1371/journal.pone.0228297)
Supplement: Attachment — Submitted filename: Response to Reviewers.docx [file pone.0228297.s005.docx]

Reviewer #1

We thank the reviewer for the comments as they increase the quality of this manuscript. We appreciate that they find it to be well written, and believe it is made better so after review.

*“The authors detail the findings from well-designed randomized cross-over trial that sought to examine the impact of prolonged standing versus prolonged sitting on energy in normal weight, healthy younger adults. The investigation of an extreme period (~12 hrs) of standing (as opposed to sitting), is a point of difference from previous investigations that have examined intermittent periods of shorter-duration standing in a fixed position. The manuscript is very well written and the authors are to be commended for the rigorous scientific methods applied.*

*The rationale for the study is well articulated, covering the current evidence that exists on this topic and what evidence gap this study sought to address.”*

*“Some aspects of the manuscript that warrant further attention include:”*

*1.” Methods: Recreationally active, but untrained participants were recruited – what screening processes were undertaken to ascertain current activity status?”*
Physical activity was ascertained via the health history questionnaire that was filled out prior to any testing. The health history questionnaire has been changed to health history and physical activity questionnaire in the manuscript for clarity. Individuals participated in some form of physical activity several times per week, but were not athletes training for any competition or event. This concept and wording is consistent with other papers in the field from our laboratory including Akins et al, 2019 and Kim et al., 2017.

*2. “Methods: It would be helpful to have additional description on what tasks the participants performed during the sitting/standing conditions? Were they able to work on a computer? How was this standardized between the two conditions?”*
Subjects were allowed to read, work on a computer, or watch television/movies. This has been added to the manuscript for clarity. Subjects were allowed to do these tasks for both trials. Subjects were not allowed to sleep during any intervention or HFGTT.

*3.” Results: A significant difference was observed for the AUCT but not the AUCI for triglycerides. For the latter, the p value was 0.186. Is it possible that this reflects a statistical power issue – ie: if the sample size was larger, could it be possible that significant differences may have been observed?”*
This is possible. Our power analysis determined we required a minimum of 12 subjects for an alpha value of 0.05 with a beta value of 0.2. Because we had 18 subjects, we felt it best to not speculate. However, it is possible that this number may become significant with more subjects, adding additional strength to the findings.

*4. “Discussion: The authors have not addressed some of the key limitations of the study, specifically the restricted generalisabilty of the study since only healthy, generally normal weight younger adults were examined. This leads one to query whether similar findings are likely to be different in middle/older-aged adults and those who are overweight?”*
This concern has been added to the paragraph addressing limitations to the study. “the subjects of this study were young healthy adults, and it is possible that these findings do not apply younger, older, or diseased populations.”

*5. “Discussion: Greater acknowledgement of the other potential detrimental consequences of extreme periods of prolonged standing (and also extreme periods of sitting for that matter) beyond that of metabolism needs to be given. For example the deleterious consequences on vascular physiology need to be acknowledged.”*
Two sentences were added to the limitations paragraph to emphasize that this study was not able to analyze other problems associated with prolonged standing including vascular dysfunction, increased heart rate variability, and lower back pain. “this study only focused on one of the detrimental outcomes to prolonged sedentary behavior. Prolonged sitting is associated with problems including endothelial dysfunction, sleeping heart rate variability, and lower back pain, but these factors are not considered in this analysis (43, 44, 45, 46).”

*6.” Discussion: The authors provide comparisons between the small changes observed in this trial with changes that have been observed with high intensity exercise. However, in the absence of study designs that have provided a direct head-to-head comparison, the comparability of the findings remains speculative.”*
This is absolutely true, and to highlight this idea, clarification has been added to the fourth paragraph of the discussion to emphasize that these comparisons are merely speculative. “While this study did not directly compare the benefits of standing to light physical activity or exercise, speculative comparisons may be drawn from other studies.”

*7. “Discussion: as acknowledged by the authors, an extreme bout of standing (and sitting) was used in this study. It would be helpful to have some further narrative on the extent to which this approach is ecologically valid and to speculate on what might occur with shorter (more ecologically valid) periods of sitting/standing.”*
The sentence that emphasized that this was an extreme, and not an ecologically valid approach was moved to an earlier part of the discussion to sooner clarify that these results are not translatable to possible lifestyle interventions. A sentence is reworded to cite that a more ecologically valid approach of alternating sitting with standing has not shown appreciable benefits to postprandial triglycerides. “More ecologically valid lengths of standing time have not shown improvements to postprandial triglyceride concentrations (14), but typically, a bout of even low intensity exercise compared to sitting, improves post-prandial metabolism the next day in subjects who are physically active (7, 18)”.

Reviewer #2

We thank the reviewer for the insightful comments that improve the manuscript and interpretation of the study.

*“This study examined the effects of posture by comparing one day of sitting to one day of standing on postprandial metabolism the following day. Despite a low ecological validity, because it is not feasible to ask people to be standing 12 hours a day, the verified results are interesting and clinically relevant, adding value for future studies of sedentary behaviour, with a reinforcing effect on the necessity of more active lifestyle as a tool for a good health.”*

“*Line 69: Why not the same period of time for both body position? Lines 117 – 118 are stated 12 hours for each behavior. Please, justify”*
This is because the total amount of sitting was 2 hours greater when including the morning and night before/after the intervention, but lines 117-118 were referring to the intervention itself.

*“Line 74- The term “recreationally active” means that the whole sample complied with physical activity recommendation for adults? Please make a clear definition on sample physical activity level!”*
This refers to the fact that all subjects engaged in some form of physical activity several times per week, but were not athletes training for any competition or event. This wording is consistent with other papers in the field from our laboratory including Akins et al, 2019 and Kim et al., 2017.

*“Line 90 – Refrain from exercise was the only recommendation? What about caffeine and alcohol ingestion?”*
Subjects were also asked to refrain from alcohol and caffeine consumption, which was confirmed through their food logs during the controlled activity phase. This has now been added to the manuscript. “Subjects were asked to refrain from exercise or consuming caffeine or alcohol from the start of the first controlled activity day to the end of the HFGTT (Fig 1)”. “Subjects were asked to consume their regular diet, sans caffeine and alcohol, and log the time and contents of everything consumed into a provided food journal”.

*“Line 98 – Metabolic rates procedures are not adequately described. Please describe a timeline and sequence of events before the indirect calorimetry procedure. This is really important because the outcomes can be affected if the sample does not comply with all recommendations.”*
Details were added to this paragraph to better clarify the procedure of gas analyzed for metabolism including a more clear description of subjects resting in the seated or standing position while breathing normally prior to gas collection.
“Each subject came to the laboratory for preliminary testing the morning after an 8-h fast. Subjects’ weight and height were measured, then each subject sat for 30 minutes then stood for 30 minutes. Subjects were asked to relax and breathe normally while sitting or standing. Subjects rested for 20 minutes before gas samples were collected to allow for a steady state to be reached (15). During the last ten minutes of each 30-minute period each subject breathed into a meteorological balloon using a one-way valve (Hans Rudolph, Kansas City, MO). Expired gas samples were analyzed with a mass spectrometer (Perkin-Elmer MGA 1100, St Louis, Missouri) for O2, CO2, and N2 concentration and a spirometer (VacuMed, Ventura, CA) for volume. The data from these gas samples were used to determine caloric expenditure during sample collection using standard formulas for indirect calorimetry, which was extrapolated to estimate resting and standing metabolic rate (16). This calculation of resting metabolic rate was used to determine the caloric content of the meals provided during the intervention phase.”

*“The Steady state was considered in gas exchange analysis?”*
Subjects were resting in the standing or sitting position for 20 minutes prior to gas collection to bring the subject to steady state. A reference to Popp et al., 2016 has been added for steady state clarification. “Subjects were asked to relax and breathe normally while sitting or standing. Subjects rested for 20 minutes before gas samples were collected to allow for a steady state to be reached (15)”.

*“Which coeficiente of variation (CV) was achieved?”*
Our coefficient of variation for estimated metabolic rate during sitting was .175 (80.63 kcal/hour divided by standard deviation of 14.13, then multiplied by 100), and our coefficient of variation for estimated metabolic rate during standing was .184 (81.64 kcal/hour with a standard deviation of 14.99) for our resting fasted gas analysis.

*“Line 109 – What criteria was used for a valid wear time?”
How many hours a day ActivePal was used?
How many days of use?”
The inclinometer was used to identified sitting time in this phase?
What physical activity level was identified in this sample?”*
The ActivePal was secured with Tegaderm and not removed from the time it was attached to the time the subjects returned to the laboratory for the intervention phase. Subjects were provided a ‘cast cover’ to allow them to shower with the monitor on so that it needn’t be removed. The inclinometer did identify the sitting time, and the activity level was identified to match recommendations sans one subject (as discussed in the next point. The sentence ‘This monitor was not removed during the entirety of the controlled activity phase’; was added to further clarify this point of concern.”

*“All of them achieved physical activity recommendations or not?”*
Yes. One subject took over 10,000 steps, as they were required to walk because of their work, but there is no addition or removal of significance when this subject’s data are removed. Other subjects were compliant.

*“Line 217 – Change fig 4D to fig 5D”*
‘4D’ was changed to ‘5D’.

*“Line 280 - Despite a small effect of standing on metabolism in the next day, this was enough to lower fasting glucose triglyceride concentrations by 13,8%. It is important to consider that the sample were comprised by young, health, normal-weight adults. Please reframe, talk about clinical relevance.”*
The sentence was rephrased to emphasize that these findings were found in a young healthy population with clinically normal fasting plasma triglyceride concentrations. “These present data highlight that standing has a small effect on metabolism the next day, but it does lower fasting plasma triglyceride concentration by 13.8% (p=0.021) and AUCT by 11.3% (p=0.022) in a young and healthy population with clinically normal fasting plasma triglyceride concentration (29)”

*“Line 289 – Will be interesting to discuss here about LIPA benefits as well. It is not a surprise that small muscle contractions in postural activities (standing) will generate smaller metabolism alterations when compared with any exercise intensity. Following two suggested papers”.*

*Amagasa S, Machida M, Fukushima N, Kikuchi H, Takamiya T, Odagiri Y, et al. Is objectively measured light-intensity physical activity associated with health outcomes after adjustment for moderate-to-vigorous physical activity in adults? A systematic review. Int J Behav Nutr Phys Act. 2018;15(1):65. doi: 10.1186/s12966-018-0695-z.*

*Howard B, Winkler EA, Sethi P, Carson V, Ridgers ND, Salmon JO, et al. Associations of Low- and High-Intensity Light Activity with Cardiometabolic Biomarkers. Med Sci Sports Exerc. 2015;47(10):2093-101. doi: 10.1249/MSS.0000000000000631.*
We thank the reviewer for providing us with these reviews and this point. The review papers allowed us to find two articles which were cited to reference the correlations between LIPA and plasma triglyceride. This makes the paragraph more complete, with the context of light activity to high intensity exercise.

*Line 333 –“ it is important to highlight how many subjects were excluded due hyperventilation at methods section.”*
Six subjects. This has been added to the manuscript.

*“Line 345 – make clear if this small benefit has clinical relevance at this point too.”*
This sentence has been edited to emphasize that this was in a population with clinically normal fasting plasma triglyceride concentrations*.*

*Line 347 – “it is relevant to highlight here that standing it is not a more relevant strategy to blunts the increases in postprandial lipemia, but can be used to introduce changes, as an open window to achieve more active behaviors”*
The sentence has be edited to include that standing may introduce active behaviors, despite it not being as effective at reducing postprandial lipemia.

Reviewer #3

We extend our gratitude to the reviewer for the comments and efforts in making this manuscript better. We did find the comments helpful and have addressed them below.

General Comments: “Ref.: PONE-D-19-20277
“*Thank you for the opportunity to review your paper entitled: ‘Prolonged standing reduces fasting plasma triglyceride but does not influence postprandial metabolism compared to prolonged sitting’. The manuscript provides detail of a small study assessing the impact of prolonged standing on postprandial metabolic responses compared to long periods of sitting. This paper communicates original research, is reasonably well written, however there are several parts of the paper, which require some revision. I hope you find comments below helpful*.”

Specific Comments:

*“Line 46- change end of sentence to: as a modifiable risk factor…*
This sentence has been reworded as suggested.

*Line 56-57 suggest removing the sentence which assumes standing is the easiest alternative to sitting- this sentence is not referenced and there could be exceptions to this sentence (e.g. people with back pain may prefer moving to standing).*
The sentence has been reworded to consider standing as an alternative, but does not suggest that it is the easiest alternative.

*“Lines 57 to 60 have used references from studies with diverse populations (e.g. pos-menopausal groups) and some are quite old. Can I suggest you read a recent systematic review by Saunders – Acute SB and markers of Cardiometabolic mix- it may assist you to find more appropriate references.”*
A reference to a study by Thorp et al., 2014 was added to include a more diverse population for this statement. Additionally, the following sentence was reworded for clarity that nonobese adults do not see depletions to glucose concentrations either.

*“Line 63- while from an experimental point of view I can see why you would completely replace 14 hours of sitting with 12 hours of standing, I am not quite sure how this type of research would translate into the real world. There could be a number of occupational health and safety risks associated with prolonged standing, potentially breaking the standing with short periods of sitting may be more realistic. There is also some research which indicates excessive standing may impact cardiometabolic research Smith et al The Relationship Between Occupational Standing and Sitting and Incident Heart Disease. Suggest acknowledging this and then justifying your research in this regard.”*

These points are well taken. A statement regarding more ecologically valid lengths of standing has been added to the first paragraph of the discussion – “More ecologically valid lengths of standing time have not shown improvements to postprandial triglyceride concentrations”, and justification for the long period of standing was introduced earlier in the first paragraph of the discussion as later suggested.

*“Line 67 the word ‘blunts’ needs to be revised”*
The word ‘blunts’ has been changed to ‘diminishes’.

*Subjects & Methods*

*“Were the subjects required to have normal BMI?”*
Subjects were not absolutely required to have a ‘normal’ BMI for inclusion.

*“Line 75 suggest you don’t need all of the information re: mean of height and weight as this is not relevant- BMI is more relevant.”*
Height and weight have been removed from the manuscript.

*“Lines 77-78 needs rewording”*
The sentence was reworded to ‘subjects had’.

*“Line 79- suggest indicating if the study risks and procedures were communicated prior to them consenting to participate.”*
The sentence was reworded to better communicate that the subjects were informed of risks prior to consenting to participate.

*Experimental protocol*

*“Subjects undertook preliminary testing- but is doesn’t say what they were tested for.”*
This is referring to the preliminary tests described in the ‘Preliminary Testing’ section of the Experimental Protocol. The word ‘metabolic’ has been added for clarity.

*“Line 88 – replace sitting/standing with sitting or standing”*
‘sitting/standing’ was changed to ‘sitting or standing’.

*The remainder of this section is well written.*

*Results*

*“Suggest adding titles to your Figures to make them easier to interpret. With the information relating to the figures below them.”*
In an effort to remain consistent with other figures in PLOS ONE articles, figures remain untitled.

*Discussion*

*The majority of the discussion is well written.*

*“As per earlier suggestion- suggest revising the first sentence- re: natural alternative is to stand, especially given that the natural alternative would not be to stand for 12 hours straight. I acknowledge that you have recognized standing is not practical in the discussion- suggest stating this earlier in the manuscript.”*
This sentence has been revised. The term ‘natural alternative’ has been changed to ‘one possible alternative’ to reflect that it is not the preferred alternative for all populations.

*“Lines 247-250 is a good justification of your study – suggest stating this earlier.”*
This sentence was moved to be stated earlier.

*“Line 296- suggest replacing the word feeding with eating”*
‘feeding’ was changed to ‘eating’.

*“Line – 305- suggest rewording this sentence (particularly the word illuminate)”*
The sentence was reworded for clarity. ‘illuminate’ was changed to ‘decipher’, and ‘observed’ was moved towards the end of the sentence.

*“Lines 307-314- suggest adding to this section how this might affect a future study- e.g. re: assessing the effects of frequent bouts compared to constant standing”*
A sentence has been added to emphasize the potential for using these intramyocellular triglycerides in future studies with more direct measurements.

*“Note: the discussion is quite long- lines 315-328 (as LPL was not measured in the study), suggest condensing this information into a couple of sentences.”*
This paragraph was edited and shortened to be more easily digestible by the ready and take up less space.

*“Line 333- how many subjects experienced hyperventilation?”*
It was six subjects, which has been added to the manuscript.

*“Lines 345- 350- suggest more thought in this section- physical activity and exercise- describe the types of activity (LPL) or potential for breaking up sitting with bouts that would be more appropriate in the context of the workplace or where sitting is likely to occur.”*
The last half of this paragraph has been edited to emphasize that the greatest benefits are seen with high intensity exercise. It has also been added that standing may be useful to introduce more active behaviors, which is more ecologically valid and possible in the workplace than our 12 hour protocol.

*“Line 351 onwards is repetitious suggest removing it”*
The last paragraph has been removed as suggested.
